# Contribution of DNA methylation to the expression of *FCGRT* in human liver and myocardium

**DOI:** 10.1038/s41598-019-45203-1

**Published:** 2019-06-17

**Authors:** R. B. Cejas, D. C. Ferguson, A. Quiñones-Lombraña, J. E. Bard, J. G. Blanco

**Affiliations:** 1 Department of Pharmaceutical Sciences, School of Pharmacy and Pharmaceutical Sciences, The State University of New York at Buffalo, Buffalo, NY 14214 USA; 2 Genomics and Bioinformatics Core, New York State Center of Excellence in Bioinformatics and Life Sciences, The State University of New York at Buffalo, Buffalo, NY 14203 USA

**Keywords:** DNA, DNA methylation, Gene regulation, Transcriptional regulatory elements, Molecular medicine

## Abstract

FcRn mediates recycling and transcytosis of IgG and albumin in various cell types. The MHC-class-I-like protein of the FcRn heterodimer is encoded by *FCGRT*. Few determinants of variable *FCGRT* expression in humans have been identified so far. In this study, we investigated the presence of DNA methylation in regulatory regions of *FCGRT* in samples of human liver and myocardium tissue, and we examined the impact of *FCGRT* methylation on FcRn expression in model cell lines. Quantitative DNA methylation analysis of the *FCGRT* locus revealed differentially methylated regions in DNA from liver and myocardium. Methylation status in individual CpG sites correlated with *FCGRT* mRNA expression. Data from model cell lines suggest that differential methylation in the −1058 to −587 bp regulatory region of *FCGRT* contributes to FcRn expression. Chromatin immunoprecipitation assays indicate that CpG site methylation impacts the binding of the methylation sensitive transcription factors Zbtb7a and Sp1. This study provides a foundation to further define the contribution of epigenetic factors during the control of FcRn expression and IgG traffic in human tissues.

## Introduction

The human neonatal Fc receptor (FcRn) is a heterodimer comprised of a MHC-class-I-like heavy chain and beta-2-microglobulin^1^. The MHC-class-I-like protein of FcRn is encoded by the *FCGRT* gene. FcRn mediates recycling and transcytosis of IgG and albumin in various cell types^2–5^. In humans, FcRn is the only high affinity and pH specific receptor of IgG^6^. In liver, FcRn contributes to maintain homeostatic concentrations of albumin and IgG by preventing their catabolism^7^. FcRn-mediated recycling allows IgG and albumin to have half-lives in blood approaching three weeks^8,9^. FcRn is also an important determinant for the pharmacokinetic and pharmacodynamic properties of protein therapeutics that contain Fc domains such as monoclonal antibody drugs^10,11^.

Variable *FCGRT* gene expression may contribute to drive the expression of the FcRn heterodimer under normal and pathological conditions^12–14^. Few determinants of variable *FCGRT* expression in humans have been identified so far. For example, Liu *et al*. have shown that *FCGRT* expression is decreased by INF-gamma via the JAK/STAT pathway and increased by proinflammatory stimulus through intronic NF-kappaB elements^15,16^. Polymorphic variable number of tandem repeats (VNTR) in the promoter region impact FcRn expression^17^. A previous report from our group described *hsa-miR-3181* as a potential regulator of *FCGRT* expression^18^.

DNA methylation contributes to the control of gene expression through processes involving modifications of chromatin structure that impact protein-DNA interactions^19^. In general, DNA methylation is associated with gene repression, although the resulting effect on gene expression is also dependent upon the location of differentially methylated regions^20,21^. DNA methylation occurs predominantly at CpG dinucleotides, and CpG density and methylation status on regulatory regions may affect gene expression^22^. The extent of *FCGRT* methylation and its potential contribution to differential FcRn expression remains to be defined. In this study, we investigated the presence of DNA methylation in regulatory regions of *FCGRT* in samples of human liver and myocardial tissue. We examined the impact of *FCGRT* methylation on the expression of FcRn in model cell lines. Our results provide insights into the role of variable DNA methylation in *FCGRT* during the expression of FcRn.

## Results

### *FCGRT* methylation in human liver and myocardium

**Figure 1:**
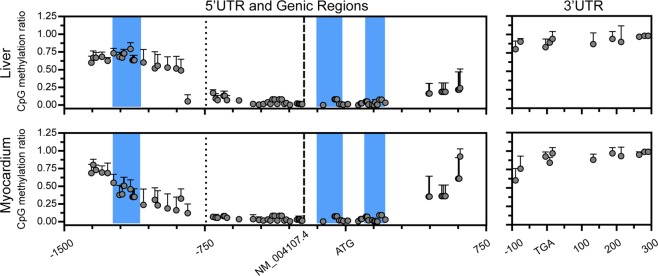
Quantitative DNA methylation analysis of the *FCGRT* locus in human tissues. Each point represents DNA methylation ratios ± SD (n = 2–10) at individual CpG sites in liver (upper panel) and myocardium (lower panel). Left panels represent the 5′UTR and partial genic regions of *FCGRT*. Right panels represent the 3′UTR of *FCGRT*. Nucleotide positions are relative to the A_+1_TG (left panel) and T_+1_GA codons (right panel), respectively. Blue-shaded regions represent CpG islands. The dotted line represents the −744 bp reference site for “distal” and “proximal” 5′UTR regions, and the dashed line represents the transcription start site for *FCGRT* transcript NM_004107.4 (−222 bp).

Bioinformatics analysis of a ≈2.0 Kb region encompassing up to 1.4 Kb upstream and 0.6 Kb downstream the start A_+1_TG codon of *FCGRT* revealed three distinct CpG islands (CGI_#1_: −1241 to −1086 bp relative to A_+1_TG, CGI_#2_: −155 to −9 bp, and CGI_#3_: +101 to +218 bp) (Fig. 1). Quantitative DNA methylation analysis was performed to examine the extent of methylation in CpG sites located within the “distal” and “proximal” 5′UTR of *FCGRT*, as well as exon 1 and intron 1, in DNA samples from human liver and myocardium (Supplementary Table S1). The analysis encompassed approximately 82% (53 sites) of the possible CpG sites located within 1.4 Kb upstream the A_+1_TG codon (Fig. 1). In samples from both tissues, DNA methylation was most pronounced at CpG sites located within the distal portion of the 5′UTR, while DNA methylation was largely absent within the proximal region (Fig. 1). On average, the extent of DNA methylation in the distal 5′UTR of *FCGRT* was 58.7 ± 12.9% in liver and 41.2 ± 9.1% in myocardium (Fig. 1). The extent of DNA methylation within the proximal 5′UTR was 4.6 ± 3.9% in liver and 4.5 ± 2.8% in myocardium (Fig. 1). DNA methylation analysis also included a total of 26 CpG sites within a 610 bp stretch downstream the A_+1_TG codon (exon 1 and intron 1) (Fig. 1). Within these genic regions the overall extent of CpG methylation was low and similar between both tissues (liver: 8.6 ± 8.3%, myocardium: 8.4 ± 10.5%) (Fig. 1). A total of 11 CpG sites located near or within the 3′UTR (−90 to + 291 bp relative to the stop codon, T_+1_GA) were analyzed and showed high levels of methylation (liver: 93.4 ± 4.4%, myocardium: 94.1 ± 5.3%) (Fig. 1).

### *FCGRT* mRNA expression in liver and myocardium

**Figure 2:**
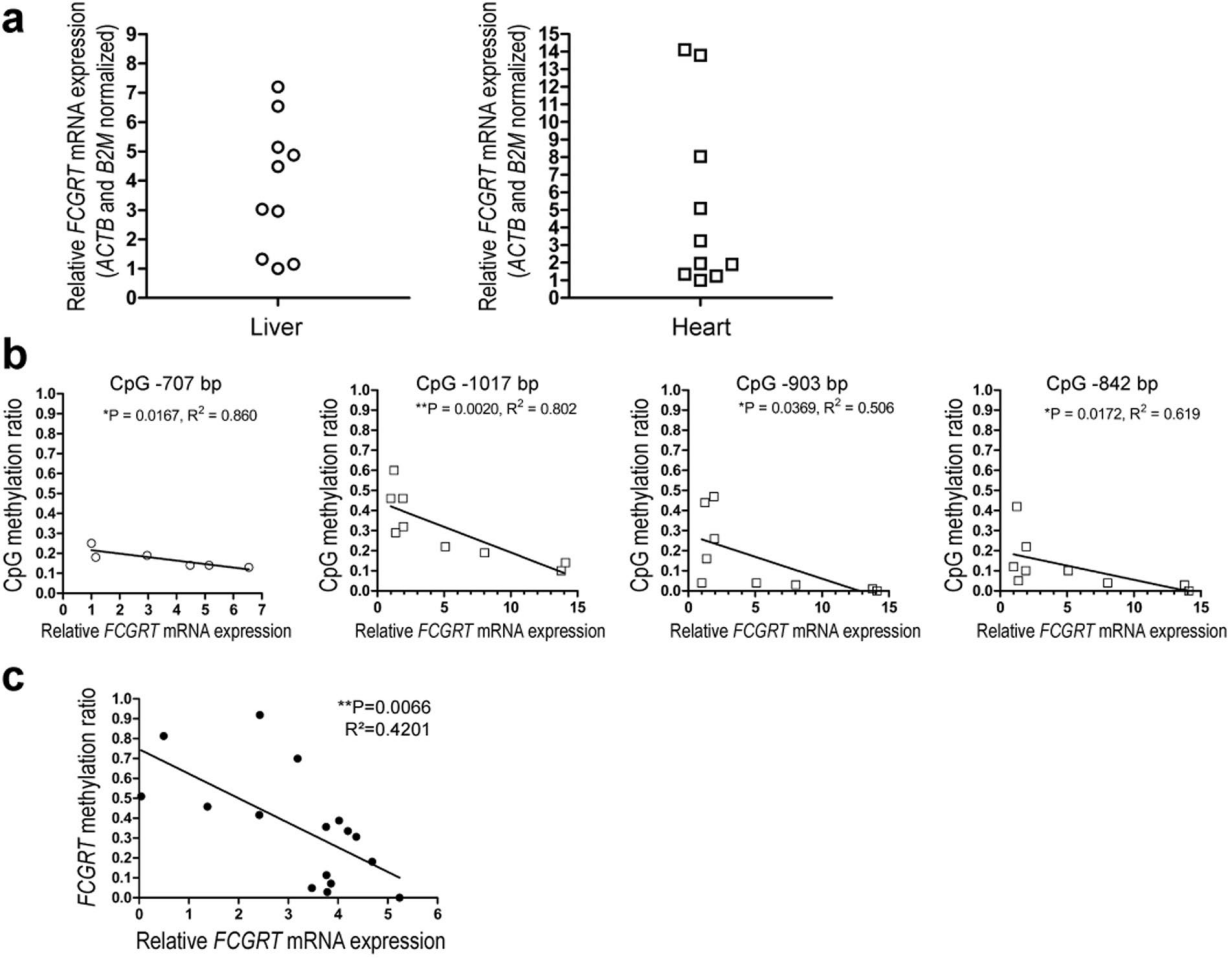
DNA methylation status and *FCGRT* expression correlation analysis in human liver and myocardium. (**a**) Relative *FCGRT* mRNA fold expression in samples from human liver (n = 10) and myocardium (n = 10). Each point represents the mean from two separate measurements performed in triplicates. (**b**) Linear regression analysis of methylation at individual CpG sites in DNA samples from liver (◦) and myocardium (▫) versus *FCGRT* mRNA relative fold expression. Detection of the −707 bp CpG site failed in 4 liver samples (◦), and detection of CpG sites −1017, −903 and −842 bp was successful in 9 out of 10 myocardial samples (▫). (**c**) Linear regression analysis of DNA methylation ratios versus *FCGRT* mRNA relative expression in liver-derived cell lines (n = 16. Data from the Cancer Cell Line Encyclopedia database). Data show mean methylation ratios in the −1021 to −628 bp region of *FCGRT*. *P < 0.05, **P < 0.01, Spearman rank-order correlation.

The expression of *FCGRT* mRNA (transcript NM_004107.4) in samples from liver and myocardium varied by approximately 7- and 14- fold, respectively (Fig. 2a). Correlation analyses between methylation levels and *FCGRT* mRNA expression were performed across all CpG sites located within the 5′UTR. In liver tissue, there was a significant negative correlation between methylation levels at CpG site −707 bp and *FCGRT* mRNA expression (Fig. 2b). In myocardium, there were negative correlations between the extent of methylation at CpG sites −1017 bp, −903 bp, and −842 bp and *FCGRT* mRNA expression (Fig. 2b). The analysis was expanded by using DNA methylation and *FGCRT* mRNA expression data from 16 liver-derived cell lines available at the Cancer Cell Line Encyclopedia (Supplementary Table S1) (CCLE, https://portals.broadinstitute.org/ccle)^23^. In liver-derived cell lines, there was a negative correlation between the extent of DNA methylation in the −1021 to −628 bp region of *FCGRT* and *FCGRT* mRNA expression (R^2^ = 0.4201, *P* = 0.0066) (Fig. 2c).

### Basal FcRn expression and *FCGRT* methylation in model human cell lines

**Figure 3:**
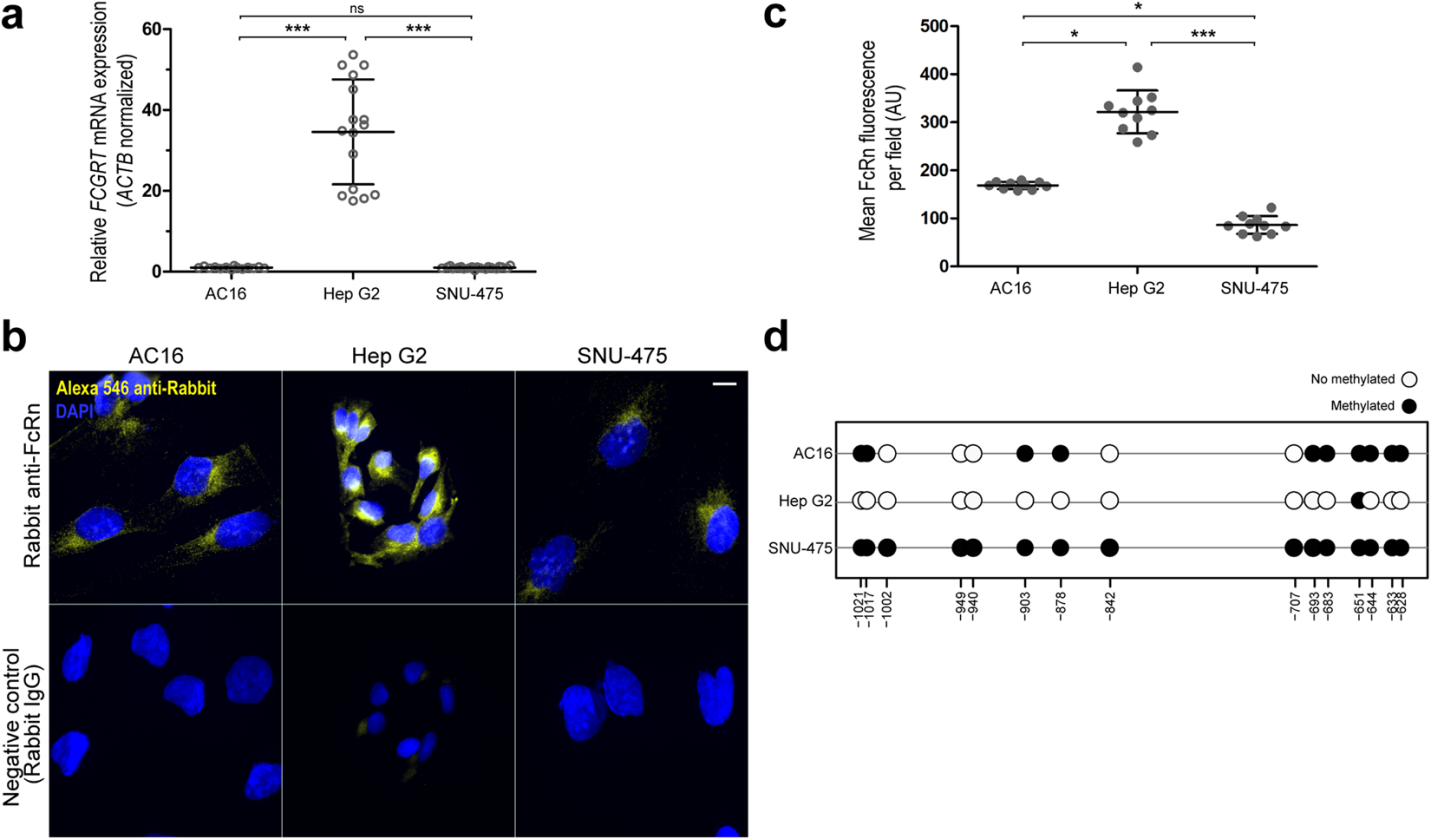
*FCGRT* expression and DNA methylation status in AC16, Hep G2, and SNU-475 cells. (**a**) Basal *FCGRT* mRNA relative fold expression. Each point represents individual measurements. Horizontal bars show the mean ± SD from three determinations performed in quintuplicates. *** P < 0.001, ns = not significant, 1-way ANOVA (Turkey’s test). (**b**) Fluorescence microcopy analysis of basal FcRn expression (yellow). Nuclei were stained with DAPI (blue). Scale bar: 10 µm. (**c**) Quantitation of FcRn relative expression. Each point represents an individual measurement. Horizontal bars show the mean ± SD (arbitrary units, AU) from a representative experiment. *** P < 0.001, ** P < 0.01 ANOVA (Kruskal-Wallis test). (**d**) Bisulfite analysis of CpG sites methylation status in the −1058 to −587 bp region of *FCGRT*. Black circles represent methylated CpG sites (≥1 positive clone), and clear circles represent demethylated CpG sites (no positive clones).

Basal expressions of *FCGRT* mRNA and FcRn protein were evaluated in the human hepatic cell lines HepG2 and SNU-475 (hepatocellular carcinoma), and in the cardiomyocyte cell line AC16 (SV40 transformed) (Fig. 3). Under basal conditions, AC16 and SNU-475 cells expressed similar *FCGRT* mRNA levels (1.00 ± 0.26 and 1.00 ± 0.29 relative fold, respectively), while HepG2 cells expressed the highest relative levels of *FCGRT* mRNA (34.59 ± 12.99 relative fold) (Fig. 3a). The trends in *FCGRT* mRNA expression were paralleled by basal FcRn protein expression. That is, HepG2 cells exhibited the highest levels of FcRn expression (245 ± 34 Arbitrary Units of fluorescence, AU) compared to AC16 (118 ± 5 AU) and SNU-475 (50 ± 11 AU) cells (Fig. 3b,c). Next, the extent of basal methylation in the *FCGRT* locus (−1058 to −587 bp region) was analyzed by bisulfite DNA sequencing. In general, there was a negative correspondence between the total number of methylated CpG sites in each cell line (i.e., SNU-475: 14 methylated/15 total CpG sites, AC16: 9 methylated/15 total CpG sites, and HepG2: 1 methylated/15 CpG sites) and basal *FCGRT* mRNA and FcRn expression (Fig. 3d).

### Impact of *FCGRT* methylation status on the expression of FcRn in model human cell lines

**Figure 4:**
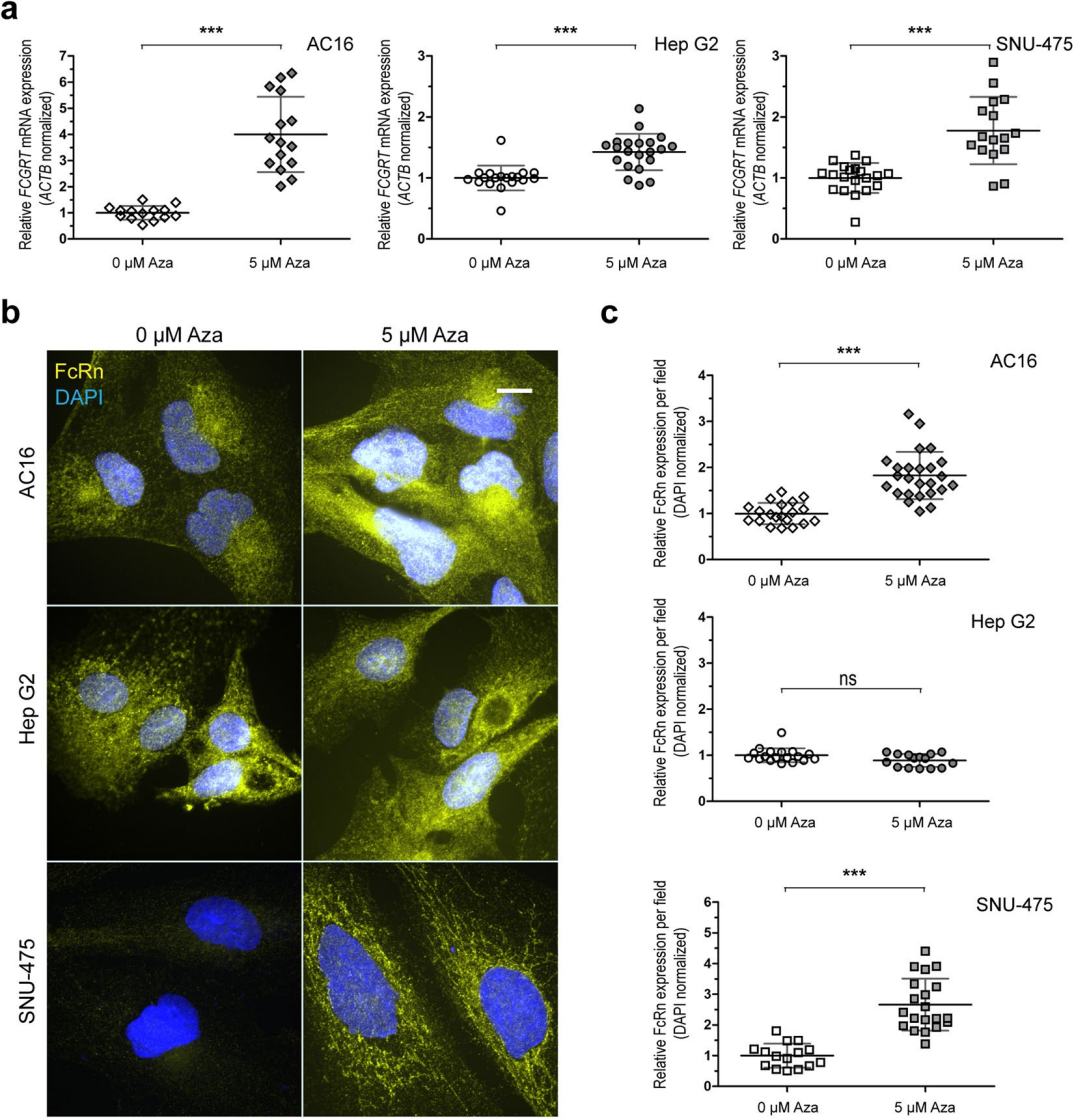
Impact of Aza treatment on *FCGRT* expression. (**a**) *FCGRT* mRNA expression in AC16, Hep G2, and SNU-475 cells after Aza treatment (5 µM, 72 h). Plots depict fold differences in *FCGRT* mRNA expression levels relative to controls (0 µM Aza). Each point represents individual measurements. Horizontal bars show the mean ± SD from three experiments performed in quintuplicates. *** P < 0.001, ns = not significant, Student’s t test. (**b**) Cellular FcRn protein expression (yellow) before and after Aza treatment (5 µM, 72 h. Representative images). Nuclei were stained with DAPI (blue). Scale bar: 10 µm. (**c**) Quantitation of FcRn relative fold expression after Aza treatments. Each point represents individual measurements. Horizontal bars show the mean ± SD from two independent experiments (8–10 fields per condition). *** P < 0.001, ns = not significant, Student’s t test.

**Figure 5:**
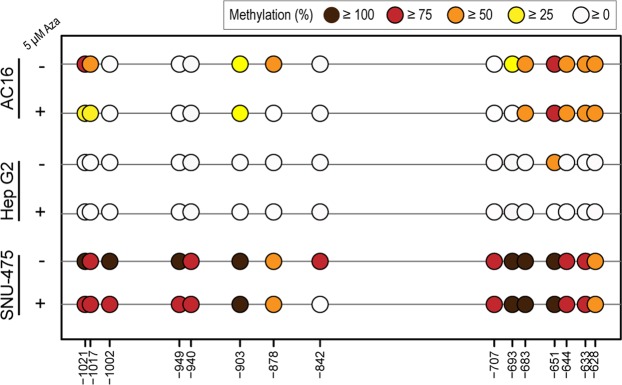
Effect of Aza treatment on *FCGRT* DNA methylation. Methylation levels at individual CpG sites in the −1058 to −587 bp region of *FCGRT* after Aza treatment (5 µM, 72 h). Circles represent individual CpG sites. Mean methylation level (%) at individual CpG sites were determined by bisulfite sequencing.

The impact of *FCGRT* gene methylation on the dynamics of FcRn expression was examined by treating HepG2, SNU-475, and AC16 cells with the demethylating drug 5-Aza-2′-deoxycytidine (Aza). Treatments with 5 µM Aza for 72 hours exerted negligible cytotoxicity in all cell lines (Supplementary Fig. S1). Aza treatment increased *FCGRT* mRNA expression in all cell lines (i.e., SNU-475: 80%, AC16: 300%, and HepG2: 40%) (Fig. 4a). Fluorescence microscopy analysis showed that Aza treatments increased FcRn protein expression in SNU-475 cells (166%) and AC16 cells (80%), but not in HepG2 cells (Fig. 4b,c). Aza treatment modified CpG methylation status in the −1058 to −587 bp region of *FCGRT* (Fig. 5). In basal conditions, HepG2 cells showed the lowest levels of overall methylation in the −1058 to −587 bp region, AC16 cells displayed an intermediate degree of methylation, while SNU-475 exhibited the higher extent of constitutive CpG methylation (Table 1). Analysis of the extent of DNA demethylation after Aza treatment showed that in general, the *FCGRT* region including CpG sites from −1021 to −842 bp was more sensitive to Aza treatment in SNU-475 and AC16 cells (Table 1). Specific CpG sites (i.e., AC16: −1021 bp and −878 bp, and SNU-475: −842 bp) were relatively more sensitive to the de-methylating effect of Aza (Fig. 5). Aza treatment for 120 h resulted in more pronounced changes in CpG methylation status and decreased cellular viability (Table 1, Supplementary Fig. S1).

**Table 1:** DNA methylation (%) in the *FCGRT* locus before and after Aza treatment.

Cell line	CpG sites in −1021 to −842 bp region			CpG sites in −707 to −628 bp region			CpG sites in −1021 to −628 bp region		
	Basal	5 µM Aza 72 h	5 µM Aza 120 h	Basal	5 µM Aza 72 h	5 µM Aza 120 h	Basal	5 µM Aza 72 h	5 µM Aza 120 h
AC16	25 ± 28%	9 ± 12%	0 ± 0%	46 ± 22%	41 ± 27%	21 ± 9%	35 ± 27%	24 ± 26%	10 ± 12%
Hep G2	0 ± 0%	0 ± 0%	0 ± 0%	7 ± 17%	0 ± 0%	0 ± 0%	3 ± 12%	0 ± 0%	0 ± 0%
SNU-475	84 ± 17%	69 ± 27%	0 ± 0%	80 ± 19%	82 ± 19%	32 ± 17%	83 ± 18%	75 ± 25%	15 ± 20%

### Binding of transcription factors to the 5′UTR of *FCGRT*

**Figure 6:**
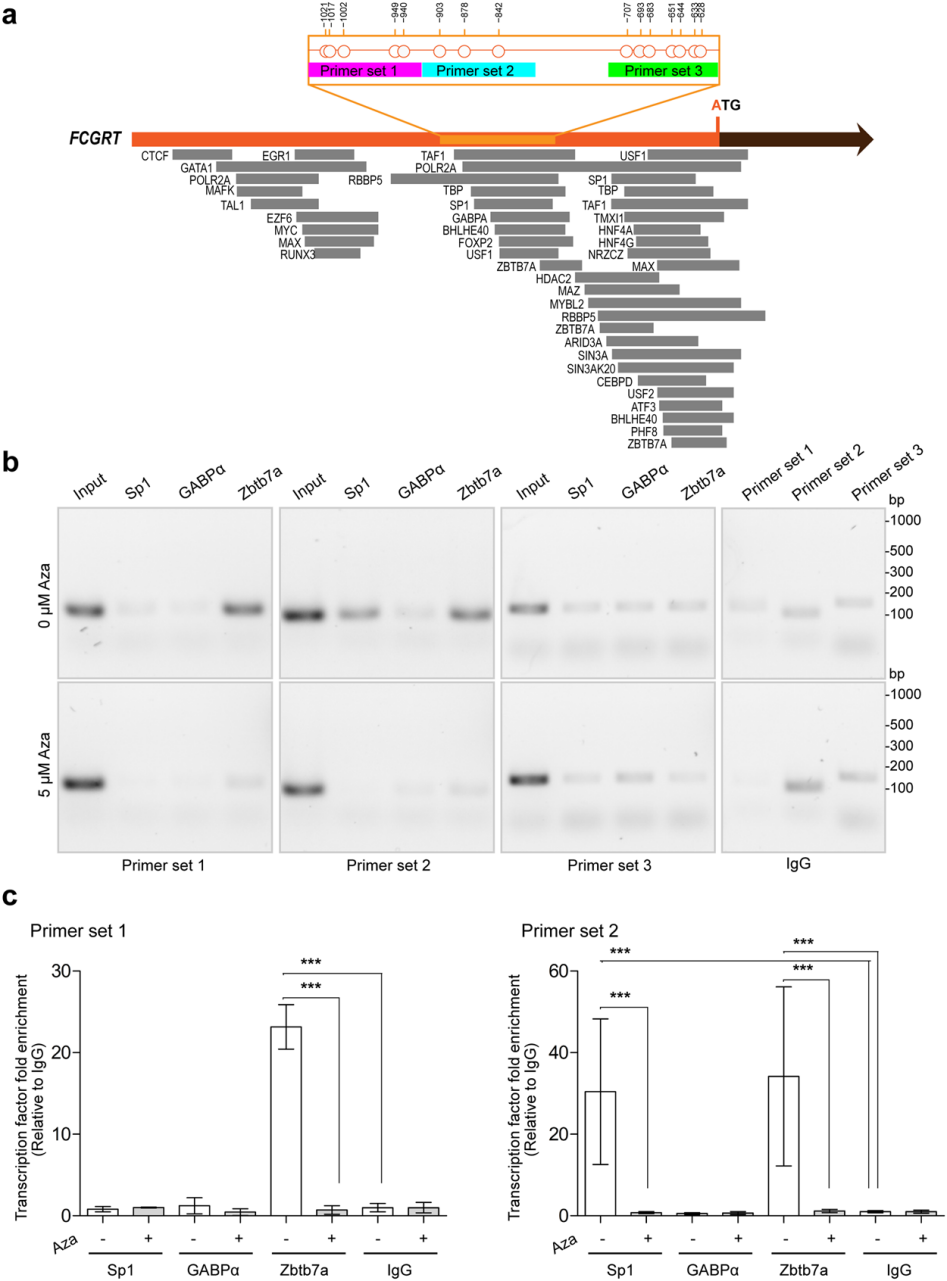
Role of DNA methylation on the binding of transcription factors to *FCGRT*. (**a**) Bioinformatics analysis of transcription factors potentially targeting the *FCGRT* locus (−1793 bp to A_+1_TG region). The −1058 bp to A_+1_TG region is shown in orange. Regions amplified by primer sets 1, 2, and 3 are indicated in magenta, cyan and green, respectively. CpG sites are represented as circles. (**b**) Chromatin immunoprecipitation (ChIP) assays evaluating the binding of Sp1, GABPα, and Zbtb7a, to the *FCGRT* locus in AC16 cells with or without Aza treatment (5 µM, 72 h). Panels show representative agarose gel electrophoresis analysis of PCR amplification products obtained with primers sets 1–3 using input samples and immunoprecipitated DNA samples. IgG controls are also shown (right panels, top, and bottom). Images were cropped from pictures taken from 3 individual agarose gels (full length pictures are included in supplementary material file 1, Figure S3). (**c**) Quantitative PCR analysis of ChIP samples assessing transcription factor enrichment in *FCGRT* regions amplified by primer sets 1 and 2. Values are expressed relative to values from IgG control antibody. Data show the mean ± SD from two independent qPCR experiments (3–4 replicates). *** P < 0.0001, 1-way ANOVA (Turkey’s test).

Bioinformatics analysis identified multiple potential binding sites for transcription factors to the 5′UTR of *FCGRT* (Fig. 6a). The region with differentially methylated CpG sites (i.e., −1058 to −587 bp) contained potential binding sites for Sp1 transcription factor (Sp1), GA-binding protein alpha chain (GABPα), and zinc finger and BTB domain-containing protein 7A (Zbtb7a) (Fig. 6a). These transcription factors were selected for further analysis based on the following criteria: 1) ubiquitous expression according to the human protein atlas database (http://www.proteinatlas.org), 2) potential binding to the *FCGRT* target region according to MeDReaders (http://medreader.org), and 3) previous evidence of interactions with the *FCGRT* promoter region^24–27^. AC16 cardiomyocytes were selected to analyze the role of *FCGRT* methylation on the binding of candidate transcription factors because the cells: 1) display intermediate level of *FCGRT* methylation, and 2) are sensitive to Aza treatment. In AC16 cells, Sp1 and Zbtb7a bind to the 5′UTR of *FCGRT* as suggested by chromatin immunoprecipitation experiments (ChIP), while GABPα showed no apparent binding to the same region (Fig. 6b,c). ChIP assays with primers spanning differentially methylated segments evidenced interactions between Sp1 and the −923 to −825 bp region (30 ± 17-fold enrichment, primer set 2), and Zbtb7a and the −1045 to −825 bp region of *FCGRT* (23 ± 3-fold enrichment, primer set 1, and 34 ± 22-fold enrichment, primer set 2) (Fig. 6c). No relevant interactions between any of the three transcription factors and the −729 to −592 bp region (primer set 3) were detected (Fig. 6b). No interactions between GABPα, Sp1, or Zbtb7a, and the −1045 to −825 bp region were evident after treating AC16 cells with Aza (5 µM for 72 h) (Fig. 6b,c). Confirmatory dot-blot assays showed that GABPα, Sp1, and Zbtb7a were expressed in AC16 cells under basal conditions and after incubations with Aza (Supplementary Fig. S2).

## Discussion

In this study, we investigated the role of DNA methylation as potential epigenetic regulator of human *FCGRT* expression. Quantitative DNA methylation analysis of the *FCGRT* locus revealed differentially methylated regions in DNA samples from liver and myocardium (Fig. 1). Seminal work by Mikulska and Simister showed that the −1529 to −570 bp region of *FCGRT* is essential for gene promoter activity^26^. This region exhibits variable levels of CpG methylation, and the extent of methylation in certain CpG sites (i.e., CpG sites: −1017, −903, −842, and −707 bp) correlates with *FCGRT* mRNA expression in liver and myocardial tissues (Fig. 2a,b). In line, global methylation levels in the −1529 to −570 bp region correlates with *FCGRT* mRNA expression in cell lines derived from hepatocellular carcinomas (Fig. 2c).

In general, increased DNA methylation in gene regulatory regions decreases gene expression^28,29^. HepG2 cells exhibited low levels of *FCGRT* locus methylation and relatively higher levels of basal *FCGRT* mRNA and FcRn protein expression in comparison to AC16 and SNU-475 cells (Fig. 3). Treatments with the de-methylating drug Aza decreased the levels of *FCGRT* locus methylation in AC16 and SNU-475 cells which was paralleled by increases in *FCGRT* mRNA and FcRn expression (Figs 4 and 5). Thus, data from these model cell lines further suggest that differential methylation in the −1058 to −587 bp regulatory region of *FCGRT* contributes to FcRn expression in liver and myocardium. Liver and myocardium are tissues composed of different cell types that exhibit variable levels of FcRn expression. For example, Kupffer cells from human liver express relatively high levels of FcRn in comparison to sinusoidal endothelial cells^30^. Thus, future studies should aim to elucidate the contribution of differential *FCGRT* methylation to the dynamics of FcRn expression in individual cell types from relevant tissues. Furthermore, epigenetically active substances such as 5-azacytidine and 5-aza-2′ deoxycytidine are used in the clinic for the therapy of myelodysplastic syndromes (MDS) and acute myeloid leukemia (AML)^31,32^. Since Aza increases the expression of FcRn in model cell lines, it will be of interest to evaluate whether Aza treatment modifies a) the pharmacological behavior (e.g., pharmacokinetics) of Fc-containing monoclonal antibody drugs, and b) albumin tumor consumption in the context of therapy for AML/MDS^33,34^.

Relatively few regulatory elements within the putative promoter region of human *FCGRT* have been characterized to date. Sachs *et al*. demonstrated that a polymorphic VNTR in the *FCGRT* promoter influences FcRn expression^17^. Mikulska showed that Sp1, Sp2, Sp3, c-Fos, c-Jun, YY1, C/EBPβ and C/EBPΔ interact with distinct regions in the *FCGRT* promoter^26,35^. Our results in AC16 cells suggest that Zbtb7a and Sp1 bind to overlapping methylated regions in the *FCGRT* promoter (Fig. 6). Zbtb7a and Sp1 interactions with *FCGRT* were abolished with Aza treatment (Fig. 6). Zbtb7a recognizes methylated CpGs and represses transcription by mechanisms that include prevention of transcription factor binding to DNA and recruitment of corepressors and histone deacetylase (HDAC) complexes^36–38^. Sp1 can recognize methylated CpG sites, and Zbtb7a is capable of interacting with Sp1 which results in transcriptional repression due to interference of Sp1’s DNA binding activity^37,39,40^. Thus, ChIP data may reflect the presence of a Sp1-Zbtb7a complex bound to the −923 to −825 bp region of *FCGRT*. This region is relatively more sensitive to demethylation by Aza in cells. Interindividual levels of methylation in liver and myocardium are also more variable in this region and this variability may be relevant for the epigenetic control of *FCGRT* expression. Additional studies are necessary in order to elucidate the role of *FCGRT* methylation on the binding of Zbtb7a, Sp1, and other transcription factors in various cell types and tissues at different diseases states.

Our results suggest that differential DNA methylation in specific regions of the *FCGRT* gene promoter contributes to regulate the expression of FcRn. This study provides a foundation to further define the contribution of epigenetic factors during the control of FcRn expression and IgG traffic in human tissues.

## Methods

### Human tissue samples

The Institutional Review Board of the State University of New York at Buffalo (UB-IRB) approved this research. UB-IRB determined that this research is not research with human subjects. This research meets exempt criteria, 45 CRF 46,101(b)(4). Human liver (n = 10) and myocardial (n = 10) tissue samples were provided by the National Disease Research Interchange (NDRI), the Cooperative Human Tissue Network (CHTN), and the Liver Tissue Procurement and Distribution System (LTPDS, National Institutes of Health Contract N01-DK-9-2310). As per Federal and State regulations, CHTN, NDRI, and LTPDS require every procurement site to obtain informed consent in compliance with all regulations governing that process in writing from any donor of human tissue (or the next of kin thereof) for the use of tissue for research. Each signed consent form is kept on file at the tissue acquisition site and information is never released to any third party. CHTN, NDRI, and LTPDS assign computer generated codes to each donor and do not maintain any information that could be used to identify the donor. CHTN, NDRI, and LTPDS provide anonymous samples coded with unique sample identification numbers. Procurement protocols for this project were reviewed and approved by NDRI, CHTN, and LTPDS.

### Cell culture and 5-Aza-2′-deoxycytidine treatments

AC16 (Millipore Sigma), HepG2 (American Type Culture Collection, ATCC), and SNU-475 (ATCC) were cultured using DMEM/F12, DMEM, and RPMI, respectively (Life Technologies), supplemented with 10% (v/v) fetal bovine serum (FBS) in standard incubation conditions at 37 °C, 5% CO_2_, and 95% relative humidity.

Cells were seeded 24 h before 5-Aza-2′-deoxycytidine (Aza, Sigma-Aldrich) treatments. Aza was added to fresh culture media every 24 h for a total of 72 h. Cell viability was determined with the CellTiter-Glo Luminescent Viability Kit (Promega), according to the manufacturer’s instructions.

### DNA methylation analysis

Genomic DNA from tissues was extracted using the E.Z.N.A. Tissue DNA kit (Omega Bio-tek). Quantitative DNA methylation analysis of the *FCGRT* locus was performed with the EpiTYPER Mass Array System (Agena Bioscience) at the Genomics Shared Resource at Roswell Park Comprehensive Cancer Center (Buffalo, New York). Specific primer sets were used for amplification (Table S1). For each amplicon, standards consisting of 0%, 50%, and 100% methylated DNA were used as calibrators. The relative location of individual CpG sites was determined by using the translation start site A_+1_TG of exon 1 as the origin. The relative location of CpG sites in the 3′UTR of *FCGRT* was determined by using the stop codon (T_+1_GA) as the origin.

### Quantitative real-time polymerase chain reaction

Total RNA was isolated from cells using Trizol reagent following the manufacturer’s instructions (Thermo Fisher). *FCGRT* mRNA expression was analyzed with specific primers (Table S2). Total RNA (12.5 ng) was reverse transcribed and amplified with the iTaq Universal SYBR Green One-Step Kit (Bio-Rad). *FCGRT* and reference genes (*ACTB* and *B2M*) were amplified in parallel in a CFX96 Touch Real-Time PCR Detection System (Bio-Rad) with the following cycling parameters: 50 °C for 10 min (reverse transcription), 95 °C for 1 min, followed by 44 cycles of 95 °C for 10 s, 60.5 °C for 20 s. Calibration curves were prepared to analyze linearity and PCR efficiency. qRT-PCR data were analyzed using the ΔΔCt method with CFX manager Software (Bio-Rad). The ΔCt method was utilized for determining the relative abundance of *FCGRT* mRNA.

### Fluorescence microscopy

Cells were grown on glass coverslips, fixed in 4% paraformaldehyde in phosphate-buffered saline (PBS) for 20 min at 4 °C and permeabilized with 0.1% Triton X-100 and 200 mM glycine in PBS for 2 min at 4 °C, and washed with PBS. Samples were blocked in 3% bovine serum albumin (BSA)-PBS for 60 min at room temperature and incubated with rabbit anti-FcRn antibody (1/100, ab193148, Abcam) 2 h at room temperature followed by Alexa 546-conjugated anti-rabbit IgG (1/1,000; Invitrogen) for 1 h. Negative controls were performed by replacing the anti-FcRn antibody with rabbit IgG (ab171870, Abcam) at the same concentration during immunostaining. Controls for secondary antibody immunostaining specificity without primary antibody were also included. Nuclei were stained with DAPI. Samples were mounted onto glass slides using FluorSave (Calbiochem). Images (12 bits) were obtained with a monochrome digital camera (Carl Zeiss, Axiocam MRm) attached to a fluorescence microscope (Carl Zeiss, Axiovert 200 M) using PlanApoN 60 × 1.40 NA oil immersion objective. Identical microscope configuration and camera settings were maintained during image acquisition for conditions from the same experiment. Images from multiple fields (~10 fields/condition) were taken.

For FcRn quantification, images were processed using identical parameters with the Fiji software^41^. Images corresponding to different conditions were grouped in image stacks for further processing and comparisons. Background subtraction was identical for each stack, mean fluorescence in the 546 channel (corresponding to FcRn or negative controls) was measured by thresholding with the Huang method in Fiji, and signals from negative controls were then subtracted. Mean DAPI fluorescence signal was measured in the same way.

### Bisulfite DNA sequencing

DNA from cells was extracted using the E.Z.N.A. Tissue DNA kit and converted with bisulfite using the MethylEdge Bisulfite Conversion System (Promega). Amplicons corresponding to the *FCGRT* 5′UTR were amplified using ZymoTaq PreMix (Zymo Research) and specific primers (Table S2). Amplicons were cloned into the pCR4-TOPO vector and transformed into TOP10 chemically competent *E. Coli* following the manufacturer’s instructions (Thermo Fisher Sci.). Individual clones (n = 4-8) for each amplicon were selected, and grown in LB broth supplemented with ampicillin (Sigma-Aldrich). Plasmid DNA was extracted from each clone using the PureYield Plasmid Miniprep System (Promega) and directly sequenced at the Genomics Shared Resource at the Roswell Park Comprehensive Cancer Center (Buffalo, New York).

### Chromatin immunoprecipitation

ChIP assays were performed using the ChIP-IT High Sensitivity kit (53040, Active Motif) according to the manufacturer’s instructions. Briefly, AC16 cells were cross-linked with 1% formaldehyde at room temperature for 5 min, repeatedly washed with ice-cold PBS, and lysed using a Dounce homogenizer followed by centrifugation. Chromatin was fragmented using enzymatic shearing. A fraction of the mixture of protein-DNA complex was used as “input DNA”. Sheared chromatin (1 μg) was then incubated overnight at 4 °C with 5 μg of rabbit anti-GABPα antibody (PA5-27735, Invitrogene), rabbit anti-Sp1 antibody (sc-14027-X, Santa Cruz Biotechnology), rabbit anti-Zbtb7a antibody (A300-548A, Bethyl Laboratories) or normal rabbit IgG (ab171870, Abcam). Immuno-precipitated DNA was eluted using protein G agarose beads, then the cross-linking was reversed and the DNA was purified.

Recovered DNA samples and inputs were analyzed by PCR and qPCR. Three fragments (123, 99, and 138 bp) corresponding to the *FCGRT* promoter region were amplified with primers listed in Table S2. PCR products were resolved on a 2% agarose gel stained with SYBR Safe (Thermo Fisher Scientific), and visualized under UV light. For qPCR, DNA samples were amplified with the iTaq Universal SYBR Green One-Step Kit (Bio-Rad) in a CFX96 Touch Real-Time PCR Detection System (Bio-Rad) with the following cycling parameters: 95 °C for 1 min, followed by 35 cycles of 95 °C for 30 s, 58 °C for 30 s. The amount of DNA in each sample was extrapolated from standard curves for each PCR reaction, and fold enrichment values for target DNA sequences relative to IgG controls samples were determined.

### Dot-blot assay

AC16 chromatin samples were spotted on nitrocellulose membranes (88018, Thermo Fisher Scientific) and dried at room temperature. Samples were blocked in 0.05% Tween 20–1% BSA-PBS for 30 min at room temperature in PBS, followed by incubation with anti-β-Actin (1/1000), anti-GABPα (1/250), anti-Sp1 (1/100) or anti-Zbtb7a (1/250) or without primary antibody in 0.05% Tween 20–0.1% BSA-PBS for 2 h at room temperature. Membranes were washed in 0.05% Tween 20-PBS for 5 minutes and then probed with HRP-conjugated goat anti-rabbit secondary antibody (1/2000, Santa Cruz) in 0.05% Tween 20-PBS for 30 min at room temperature. After washing, membranes were incubated during 1 min with Pierce ECL Substrate (Thermo Fisher Scientific) and imaged in a ChemiDoc XRS+ Imager (Bio Rad).

Densitometric analysis was performed with the Fiji software^41^. A region of interest (ROI) corresponding to the spotted region was manually selected, and the mean gray value was measured. Negative control signal was subtracted from all conditions and normalized to β-actin signal.

### Bioinformatics

*FCGRT* reference sequence NM_004107.4 from the National Center for Biotechnology Information (Gene ID: 2217) was used for analysis. Predictions of CpG islands were performed with MethPrimer (http://www.urogene.org/cgi-bin/methprimer/methprimer.cgi) with the following standard criteria: island size >100 bp, CG% >50 and O/E ratio >0.6^42^.

The analysis of transcription factors potentially targeting the −1793 bp to A_+1_TG in the *FCGRT* locus was performed by incorporating ChIP-Seq datasets available from the Encode Project (https://www.encodeproject.org) and datasets available in Factorbook (http://www.factorbook.org/). These datasets were aggregated and viewed in the UCSC Genome Browser (https://genome.ucsc.edu/).

### Data processing and statistical analysis

Data processing was performed with Excel 2016 (Microsoft Office). Statistical analyses were performed with GraphPad Prism version 7. The D’Agostino & Pearson omnibus normality test was used to determine the normality of data sets. Comparisons between the means of two groups were performed with the Student’s t-test or Mann-Whitney’s U test for sets with normal and non-normal distributions, respectively. ANOVA with Tukey’s or the Kruskal-Wallis test were used to analyze differences between means from multiple groups. Spearman’s rank-order test was used for correlation analyses.

## Supplementary information

SUPPLEMENTARY INFORMATION

## Data Availability

The datasets generated during the current study are available from the corresponding author upon request.
