# Supplementary material for: Contribution of DNA methylation to the expression of FCGRT in human liver and myocardium
Source: Sci Rep. 2019 Jun 17;9:8674. doi: 10.1038/s41598-019-45203-1 (PMC6572836; doi:10.1038/s41598-019-45203-1)
Supplement: Supplementary file 1 — SUPPLEMENTARY INFORMATION [file 41598_2019_45203_MOESM1_ESM.pdf]

SUPPLEMENTARY INFORMATION

Contribution of DNA methylation to the expression of *FCGRT* in human liver and myocardium.

Cejas RB, Ferguson DC, Quiñones-Lombraña A, Bard J, Blanco JG.

Table S1. Donor demographics and liver-derived cell lines from CCLE

Liver donors		Myocardium donors		Liver-derived cells	
Gender/Age	Liver disease	Gender/Age	Heart disease	Cell line	Disease
Male/29	Normal	Female/55	Normal	HEP3B217	Hepatocellular carcinoma
Female/49	Normal	Female/47	Normal	HEPG2	Hepatocellular carcinoma
Male/62	Normal	Male/42	Normal	HLF	Hepatocellular carcinoma
Male/23	Normal	Male/40	Yes	HUH1	Hepatocellular carcinoma
Male/34	Normal	Male/42	Yes	HUH6	Hepatoblastoma
Female/34	Normal	Male/47	Normal	HUH7	Hepatocellular carcinoma
Female/59	Normal	Male/53	Normal	JHH4	Hepatocellular carcinoma
Female/58	Normal	Female/38	Normal	LI7	Hepatocellular carcinoma
Male/28	Normal	Male/70	Normal	PLCPRF5	Hepatocellular carcinoma
Female/38	Normal	Male/34	Normal	SKHEP1	Hepatocellular carcinoma
				SNU182	Hepatocellular carcinoma
				SNU398	Hepatocellular carcinoma
				SNU449	Hepatocellular carcinoma
				SNU475	Hepatocellular carcinoma
				SNU878	Hepatocellular carcinoma
				SNU886	Hepatocellular carcinoma

Table S2. PCR primers

	Primer forward sequence 5'→3'	Primer reverse sequence 5'→3'	
**qRT-PCR primers**			
qRT-PCR-*FCGRT*	TCGTGGTGGGAATCGTC	CACGAAGGGAGATCCAAGGG	
qRT-PCR FCGRT transcript variant NM_004107.4 (NCBI)	CACGGGGATACAGAGGGGT	TTACTTTGAGGGTGTCTGCCG	
qRT-PCR-B-Actin	GGACTTCGAGCAAGAGATGG	AGCACTGTGTTGGCGTACAG	
qRT-PCR-B2M	TGAAGCTGACAGCATTC	CTGCTGGATGACGTGAGTAAA	
**PCR primers**			
ChIP-FCGRT Set 1	CCAGAAGATTTTGATTAACAGCG	CAAAACTGGGAAAGGAACTCG	
ChIP-FCGRT Set 2	GTCAAGAATTCCCTGAGCGG	GCCTGTTTGTTTCTCAAAACGC	
ChIP-FCGRT Set 3	GAACTGGGGTCTCCAGTCAC	CTCAGACCCAGGGTTCCAG	
**EpiTyper primers**			
EpiTyper-Set 1	GTTTGGAGTGTAGTGGTGTAATTT	TCCTACAAATACTCTTTAATATCCTTCT	Bisulfite converted
EpiTyper-Set 2	TTTTTAAAGTGTTGGGATTATAGG	AACCCAACCTATTTATTTCTCAA	Bisulfite converted
EpiTyper Set 3	GTTTGGGGTGTGTTTTGTAATTG	CTCTCTCAAACCCAAAAATCCAA	Bisulfite converted
EpiTyper Set 4	TTGGATTTTTGGGTTTGAGAG	ATCCAAACTAACAACAATACCCTC	Bisulfite converted
EpiTyper Set 5	GGAGGGTATTGTTGTTAGTTTGGAT	ACAAATAATTCCCAACCTCAAACC	Bisulfite converted
EpiTyper Set 6	TTTTTTTTGGGTATTTGTTTTATTGTAGT	CCTCAAATCTATAATCTCTTTCTCCC	Bisulfite converted
EpiTyper Set 7	TTTTTTAGTTTTTTGGATTTTTTTT	ATTCCCCTATAACAACATATTACCTATA	Bisulfite converted
**Bisulfite sequencing primers**			
FCGRT-Bisulfite Set 1	GGAATTAAGGAGGTTTTTAGAAGAT	CAAACCCTAAACCCAACCTATTTAT	Bisulfite converted
FCGRT-Bisulfite Set 2	GAGAAATAAATAGGTTGGGTT	CCTCAAACCCAAAATTCCAA	Bisulfite converted

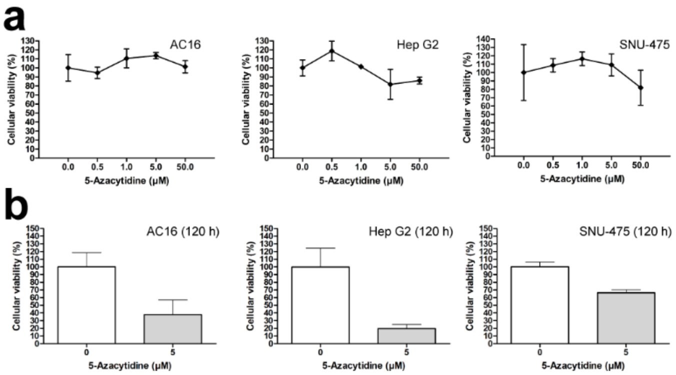

Figure S1. Effect of Aza treatments on cellular viability. a. Cellular viability evaluated after 72 h of exposure to Aza. **b.** Cellular viability after 120 h of exposure to Aza treatment (5 µM). Data represent Mean ± SD.

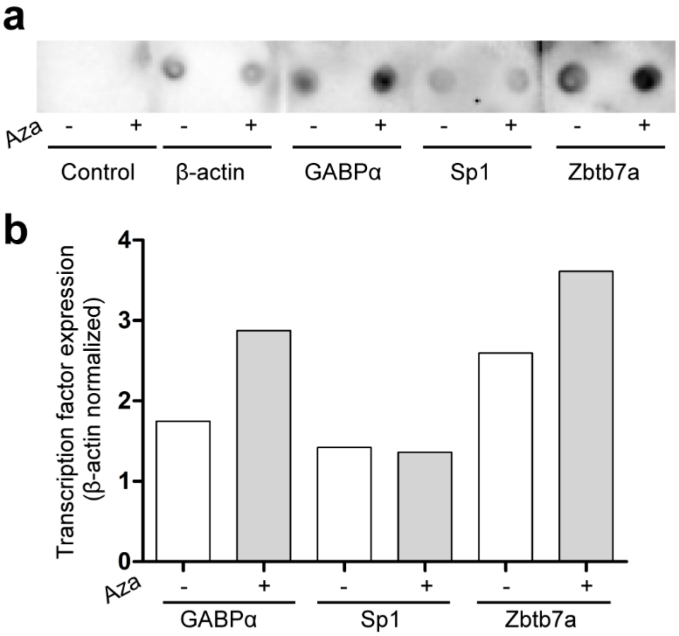

Figure S2. Expression of GABPα, Sp1 and Zbtb7a in AC16 cells treated with Aza (5 µM, 72 h). a. Representative dot blots of chromatin samples without primary antibody (control), anti-β-actin (loading control), anti-GABPα, anti-Sp1 or anti-Zbtb7a. **b.** Densitometric analysis of GABPα, Sp1 and Zbtb7a expression in AC16 cells.

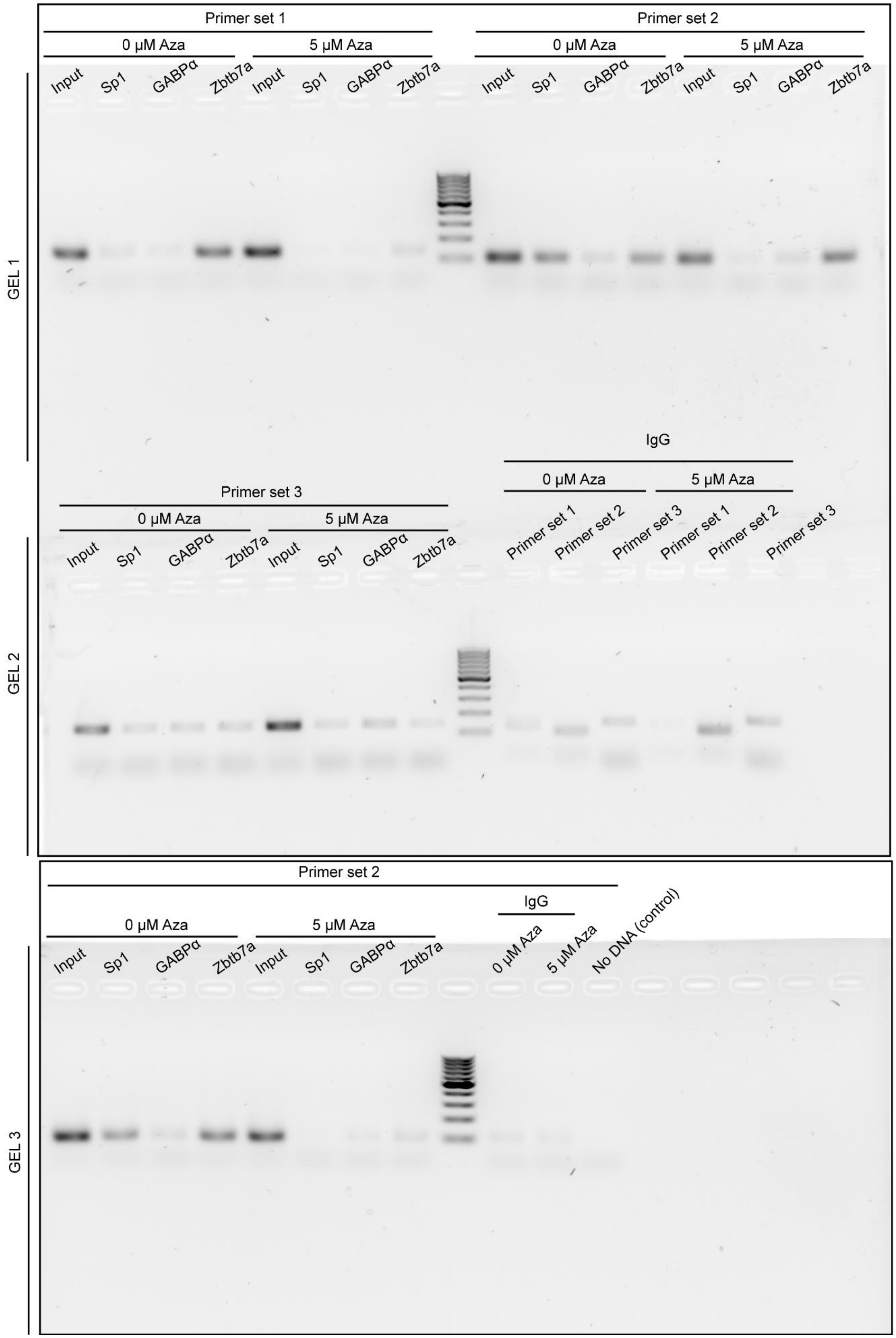
Primer set 1
0 μM Aza
5 μM Aza
Input
Sp1
GABPα
Zbtb7a
Primer set 2
GEL 1
IgG
Primer set 3
Primer set 1
Primer set 2
Primer set 3
GEL 2
GEL 3
No DNA (control)

Figure S3. Chromatin immunoprecipitation (ChIP) assays evaluating the binding of transcription factors to the *FCGRT* locus in AC16 cells with or without Aza treatment (5 μM, 72 h). Full-length agarose gels showing PCR amplification products obtained with primers sets 1 (Gel 1), 2 (Gel 1 and 3) and 3 (Gel 2) using input samples and immunoprecipitated DNA samples. Gels 1 and 2 were run and imaged under the same conditions. Gel 3 is a repetition of the analysis performed with primer set 2. See also Figure 6.
